# Supplementary material for: Transcriptional Coactivator BOB1 (OBF1, OCA-B) Modulates the Specificity of DNA Recognition by the POU-Domain Factors OCT1 and OCT2 in a Monomeric Configuration
Source: Biomolecules. 2024 Jan 17;14(1):123. doi: 10.3390/biom14010123 (PMC10812921; doi:10.3390/biom14010123)
Supplement: Supplementary file 1 [file biomolecules-14-00123-s001.zip › Figures S1-S3.pdf]

SUPPLEMENTARY FIGURES

6xHis tag – thrombin site – (BOB1) – TEV site – 3x Flag tag

6xHis tag – thrombin site – (DBD OCT1: 20 aa + POU-specific +Homeobox domain + 20 aa) – TEV site – SBP tag

6xHis tag – thrombin site – (DBD OCT2: 20 aa + POU-specific +Homeobox domain + 30 aa) – TEV site – SBP tag

Figure S1. Schematic representations of DNA cassettes inserted into the pET28a plasmid for bacterial expression of N- and C-tagged full-length BOB1 and the POU domains of OCT1 (POU1) and OCT2 (POU2). Number of amino acids and molecular weights of insertions: 6xHis tag – 6 aa, 0.8 kDa; thrombin site – 8 aa, 0.9 kDa; TEV site – 7 aa, 0.81 kDa; 3xFlag tag – 22aa, 2.7 kDa; SBP tag – 38 aa, 4.3 kDa. 6xHis tag – 6 aa, 0.8 kDa; thrombin site – 8 aa, 0.9 kDa; TEV site – 7 aa, 0.81 kDa; 3xFlag tag – 22aa, 2.7 kDa; SBP tag – 38 aa, 4.3 kDa.

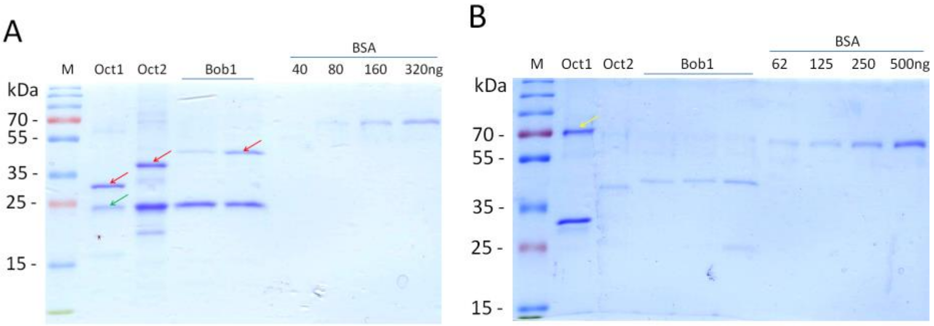

Figure S2. Analysis of bacterially expressed and purified POU1, POU2, and BOB1 proteins using SDS-PAGE (12%). (A) Separation of the indicated proteins (labeled with red arrows) after their purification on Ni–NTA His • Bind Resin (Novagen) using the N-terminal His-tag. (B) Separation of proteins after additional purification using Pierce Streptavidin Plus UltraLink Resin (POU1, POU2) or Anti-FLAG M2 Affinity Gel (BOB1) based on C-terminal tags. The additional purification step allowed to get rid of the 25-kDa protein (green arrow) co-purified in all the three samples in A. The 70-kDa band (yellow arrow) co-purified with POU1 is not related to this protein, as it does not seem to produce any complexes with DNA on EMSA (Fig. 1). The predicted molecular weights of POU1, POU2, and BOB1 are 32.1, 35.8, and 34.8 kDa, respectively. Notice the decreased electrophoretic mobility of the BOB1 protein, which does not correspond to the predicted molecular weight; similar mobility decrease was also observed for BOB1 fusion missing the C-terminal tag (data not shown).

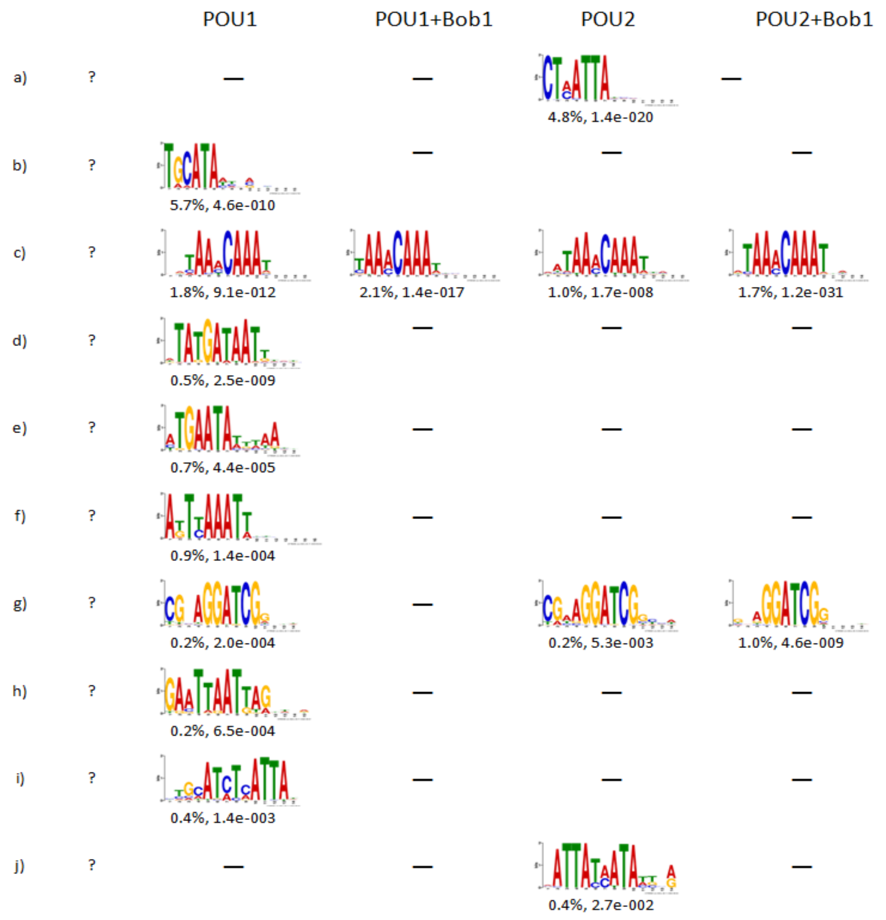

Figure S3. Minor motifs, identified by the STREME online tool, that exhibited *p*-values < 0.05 and could not be matched to any known POU-domain-binding motifs.
