# Supplementary figures and images for: Transcriptional Coactivator BOB1 (OBF1, OCA-B) Modulates the Specificity of DNA Recognition by the POU-Domain Factors OCT1 and OCT2 in a Monomeric Configuration
Source: Biomolecules. 2024 Jan 17;14(1):123. doi: 10.3390/biom14010123 (PMC10812921; doi:10.3390/biom14010123)

## Slide 1
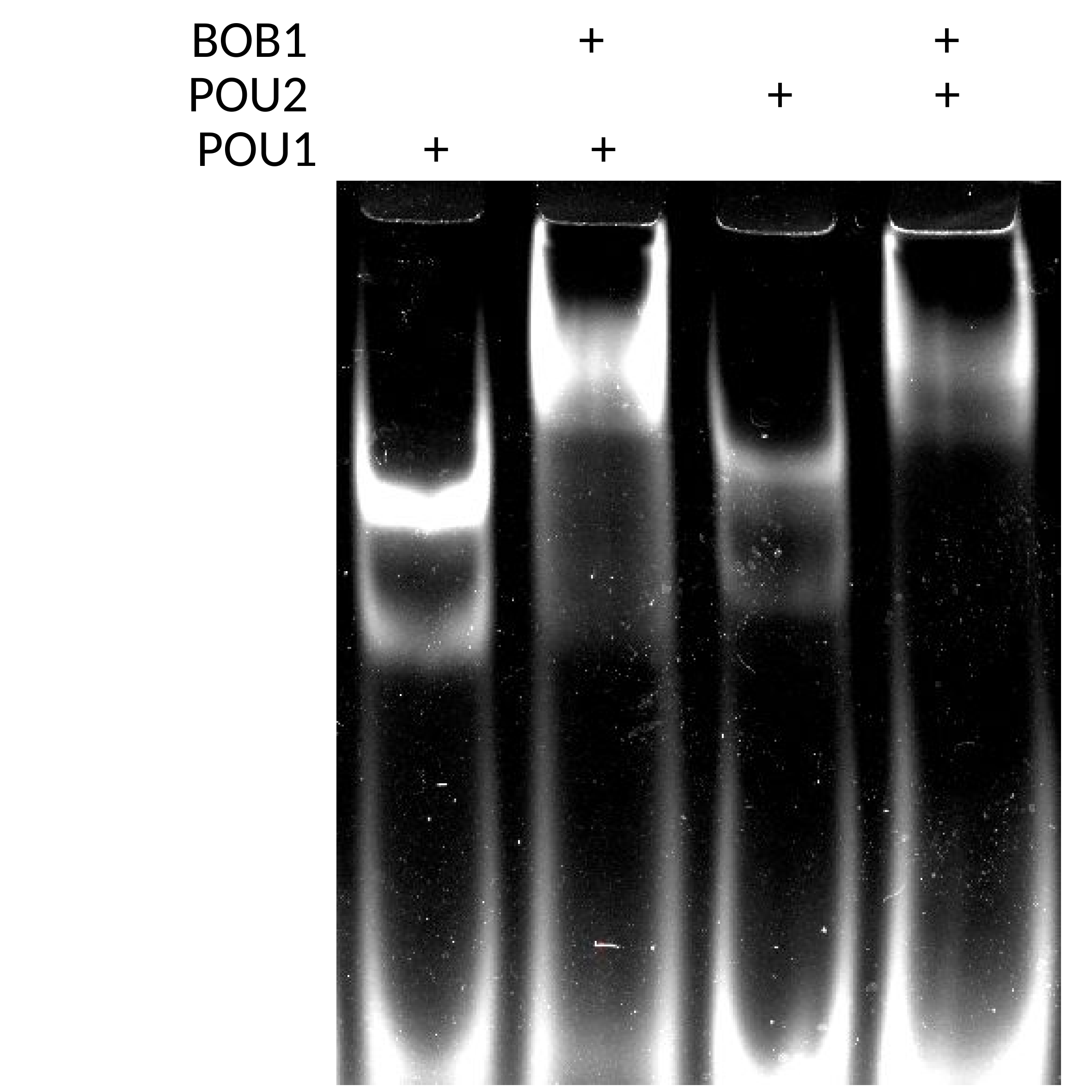

BOB1 + +
POU2 + +
POU1 + +

Supplement: Supplementary file 1 [file biomolecules-14-00123-s001.zip › File S5.pptx]

## Slide 1
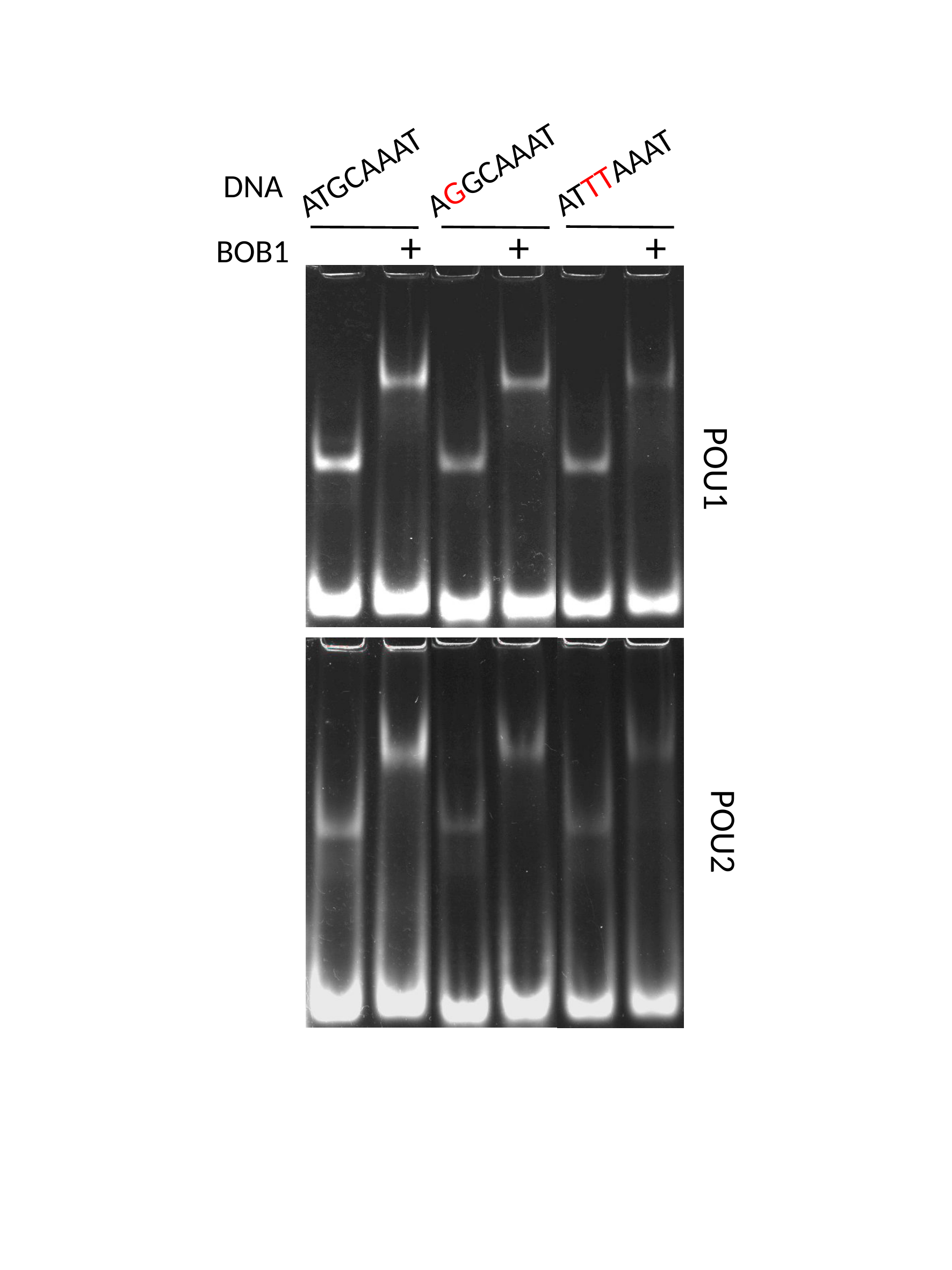

AGGCAAAT
ATGCAAAT
ATTTAAAT
 DNA
BOB1 + + +
POU1
POU2

Supplement: Supplementary file 1 [file biomolecules-14-00123-s001.zip › File S6.pptx]
